# Production of Biopolyamide Precursors 5-Amino Valeric Acid and Putrescine From Rice Straw Hydrolysate by Engineered *Corynebacterium glutamicum*

**DOI:** 10.3389/fbioe.2021.635509

**Published:** 2021-03-29

**Authors:** Keerthi Sasikumar, Silvin Hannibal, Volker F. Wendisch, K. Madhavan Nampoothiri

**Affiliations:** ^1^Microbial Processes and Technology Division (MPTD), CSIR-National Institute for Interdisciplinary Science and Technology, Thiruvananthapuram, India; ^2^Academy of Scientific and Innovative Research (AcSIR), Ghaziabad- 201002, India; ^3^Genetics of Prokaryotes, Faculty of Biology & CeBiTec, Bielefeld University, Bielefeld, Germany

**Keywords:** 5-amino valeric acid, *Corynebacterium glutamicum*, polyamides, putrescine, rice straw hydrolysate

## Abstract

The non-proteinogenic amino acid 5-amino valeric acid (5-AVA) and the diamine putrescine are potential building blocks in the bio-polyamide industry. The production of 5-AVA and putrescine using engineered *Corynebacterium glutamicum* by the co-consumption of biomass-derived sugars is an attractive strategy and an alternative to their petrochemical synthesis. In our previous work, 5-AVA production from pure xylose by C. *glutamicum* was shown by heterologously expressing *xylA* from *Xanthomonas campestris* and *xylB* from *C. glutamicum*. Apart from this AVA Xyl culture, the heterologous expression of *xylA*_*Xc*_ and *xylB*_*Cg*_ was also carried out in a putrescine producing *C. glutamicum* to engineer a PUT Xyl strain. Even though, the pure glucose (40 g L^–1^) gave the maximum product yield by both the strains, the utilization of varying combinations of pure xylose and glucose by AVA Xyl and PUT Xyl in CGXII synthetic medium was initially validated. A blend of 25 g L^–1^ of glucose and 15 g L^–1^ of xylose in CGXII medium yielded 109 ± 2 mg L^–1^ putrescine and 874 ± 1 mg L^–1^ 5-AVA after 72 h of fermentation. Subsequently, to demonstrate the utilization of biomass-derived sugars, the alkali (NaOH) pretreated-enzyme hydrolyzed rice straw containing a mixture of glucose (23.7 g L^–1^) and xylose (13.6 g L^–1^) was fermented by PUT Xyl and AVA Xyl to yield 91 ± 3 mg L^–1^ putrescine and 260 ± 2 mg L^–1^ 5-AVA, respectively, after 72 h of fermentation. To the best of our knowledge, this is the first proof of concept report on the production of 5-AVA and putrescine using rice straw hydrolysate (RSH) as the raw material.

## Introduction

The severe global environmental impact of the polyamide industries demands an alternative process to their current synthesis from the petrochemical routes. As the demand for these commercially relevant conventional plastics increases tremendously day by day, a new green approach for their sustainable production from renewable sources, with biodegradability and biocompatibility owes a competitive advantage and could be the most significant replacement for the current scenario.

Polyamides are homopolymers of terminal amino acids or co-polymers of diacids and diamines. The non-ribosomal amino acid 5-AVA is a glutaric acid derivative, an important precursor and an attractive building block for the synthesis of the polyamides. Apart from considering the importance of this five-carbon non-proteinogenic amino acid, as a potential monomer for the synthesis of nylon 5 and nylon 65 (Pukin et al., 2010; Adkins et al., 2013), their biotechnological production also gains considerable interest. A global high-performance polyamide market size report (Grand View Research Inc, 2020) projects a Compound annual growth rate (CAGR) of 7.1% for the high-performance polyamides from 2020 to 2027 as their consumption is highly increased for the production of insulation materials, industrial brushes and medical, healthcare products. 5-AVA is naturally produced as an intermediate by *Pseudomonas putida* in the degradation of L-lysine by AMV pathway (Liu et al., 2014), whereas, in *Pseudomonas aeruginosa* 5-AVA is produced by the transamination and oxidation of cadaverine, which in turn is produced by the decarboxylation of lysine (Jae et al., 2013). Successful metabolic engineering approaches have been established in *E. coli* and *Corynebacterium* strains for the production of 5-AVA from glucose, by the expression of L-lysine monoxygenase (DavB) and 5-amino valeramide amidohydrolase (DavA) genes of *Pseudomonas putida* (Cho et al., 2016). The four-carbon diamine—putrescine called 1,4-diaminobutane is a low molecular weight nitrogenous base and an essential monomer used in the chemical industry for the synthesis of effective bioplastic nylon-4,6 with a high melting point and exceptional chemical resistance. A metabolically engineered *E. coli* K12 W3110 was reported to produce putrescine from glucose minimal medium (Qian et al., 2009). The requirement for putrescine is estimated to be about 10,000 tons per year in Europe and this demand is expected to drastically increase in the coming years. Schneider et al. (2012) have reported a stable putrescine production from glucose with *C. glutamicum* by modifying the OTC activity and by expressing ornithine decarboxylase gene *sp*e*C* from *E. coli*.

The soil-dwelling Gram-positive bacterium *Corynebacterium glutamicum* has a very well-developed genetic toolbox and has been extensively explored by metabolic engineering, for decades for the effectual industrial production of amino acids such as lysine. Many value-added compounds like the *N*-methylated amino acids, sarcosine (Mindt et al., 2019), 7-choro tryptophan (Veldmann et al., 2019) and several intermediate precursors of amino acids, for example, 2-oxovalerate (Krause et al., 2010) and pyruvate (Wieschalka et al., 2012) have been successfully produced by the recombinant *C. glutamicum* strains. Several researchers across the globe have been studying the possibilities of ethanol production from the agro-residues for decades as these commodities are cheaply available in large quantities (Duff and Murray, 1996; Binod et al., 2010; Oberoi et al., 2010, 2012; Sarkar et al., 2012). The major agro-residual biomass includes rice straw, wheat straw, corn straw, cottonseed hair, seaweed, paper, pineapple leaf, banana stem, jatropha waste, and sugarcane bagasse (Kim and Dale, 2004).

Rice straw is one of the most abundant and underutilized agricultural wastes, rich in the structural carbohydrates—cellulose (32–47 %) and hemicellulose (19–27 %), densely packed in lignin (5–24 %) (Mosier et al., 2005; Kawaguchi et al., 2006). Cellulose is composed of repeating units of cellobiose and hemicellulose consists of several sugars like D-glucose, D-mannose, D-galactose, D-xylose, arabinose, and rhamnose. The depolymerization of rice straw into fermentable sugars turns it into a preferable primary carbon source for the microbial biocatalysts and biotransformation to commercially significant high-performance compounds making it an alternative to the pure sugar monomers like glucose, which have competing uses in the food industries (Jorge et al., 2016). The pretreatment of rice straw results in the formation of several compounds like furfural, hydroxyl methyl furfural, and 4-hydroxybenzaldehyde which inhibit the growth of many bacteria but *C. glutamicum* has been shown to withstand the pretreatment derived inhibitors (Gopinath et al., 2011). The wild type *C. glutamicum* ATCC 31831 utilize arabinose (Sasaki et al., 2009), whereas *C. glutamicum* ATCC 13032 consumes the pentose ribose (Wendisch, 2003; Rey et al., 2005) but cannot utilize xylose (Kawaguchi et al., 2006). *C. glutamicum* has been successfully engineered to consume xylose for growth and production. The scarcity of pentose utilizing microorganisms is a drawback for the successful industrial application of bioprocesses for the synthesis of economically important platform chemicals from lignocellulosic biomass. This study dealt with the exploitation of rice straw, a locally available surplus lignocellulosic biomass, as an alternative renewable raw material for the production of high-value products such as 5-AVA and putrescine by the simultaneous utilization of glucose and xylose, the abundant pentose sugar in biomass by employing the metabolically engineered *C. glutamicum* strains.

## Materials and Methods

### Bacterial Strains and Culture Conditions

Bacterial strains and plasmids used in this work are listed in Table 1. *Escherichia coli* DH5α containing the pECXT99A-*xylA_*Xc*_xylB_*Cg*_* plasmid (Jorge et al., 2017b) was used for extracting plasmid DNA. *E. coli* and *C. glutamicum* cells were cultivated in lysogeny broth (LB) medium (10 g L^–1^ of tryptone, 5 g L^–1^ yeast extract, and 10 g L^–1^ sodium chloride) or brain-heart-infusion (BHI) broth in 100 mL baffled flasks at 120 rpm at 37°C or 30°C, respectively. Kanamycin (25 μg mL^–1^), tetracycline (5 μg mL^–1^), spectinomycin (100 μg mL^–1^), and 1 mM isopropyl β-D-1-thiogalactopyranoside (IPTG) were added when necessary. The pre-cultures of *C. glutamicum* AVA Xyl and PUT Xyl were grown in brain-heart-infusion (BHI) broth and incubated at 30°C and 200 rpm. For fermentation experiments, *C. glutamicum* strains were grown in CGXII basal medium with desired sugar concentrations and antibiotics, incubated at 30°C, in a rotary shaker (200 rpm). For all the fermentation experiments, the seed cultures were inoculated from fresh BHI agar plates containing appropriate antibiotics and a final concentration of 1 mM isopropyl-β–D-thiogalactopyranoside (IPTG) was added at the time of inoculation. The bacterial strains were preserved in 40% glycerol at 80°C for long-term storage and maintained BHI agar plates supplemented with necessary antibiotics and preserved at 4°C for the short term.

**TABLE 1:** Strains and plasmids used in this study.

Strains and plasmids	Characteristics	References
**Strains**		
*C. glutamicum* NA6	Putrescine producer strain; *C. glutamicum* ATCC13032 *odhA^*TTG*^odhI^*T*15A^*Δ*argF*Δ*argR*Δ*snaA*, carrying IPTG-inducible pVWEx1-*speC-gapA-pyc-arg^*BA*49V/M54V^-argF_21_*	Nguyen et al., 2015
*C. glutamicum* PUT Xyl	Putrescine producer strain growing on xylose; *C. glutamicum* NA6 derivative, carrying IPTG-inducible plasmid pECXT99A-*xylA_*Xc*_xylB_*Cg*_*	This work
*C. glutamicum* AVA Xyl	5-Aminovalerate producer strain growing on xylose; *C. glutamicum* 5AVA3 (pECXT99A-*xylA_*Xc*_xylB_*Cg*_)* = GRLys1Δ*sugR*Δ*ldhA*Δ*snaA*Δ*cgmA*Δ*gabTDP* derivative carrying IPTG-inducible plasmids pVWEx1-*ldcC*, pEKEx3-*patDA*, and pECXT99A-*xylA_*Xc*_xylB_*Cg*_*	Jorge et al., 2017b
*E. coli* DH5α (pECXT99A-*xylA_*Xc*_xylB_*Cg*_*)	*E. coli* DH5α strain carrying IPTG-inducible plasmid pECXT99A-*xylA_*Xc*_xylB_*Cg*_*	Jorge et al., 2017b
**Plasmids**		
pECXT99A-*xylA_*Xc*_xylB_*Cg*_*	pECXT99A-derived (Kirchner and Tauch, 2003), IPTG-inducible vector for the simultaneous overexpression of *xylA* from *Xanthomonas campestris SCC1758 and xylB from C. glutamicum ATCC13032*	Jorge et al., 2017b

### Genetic Manipulations

*C. glutamicum* competent cells were prepared as described by Jorge et al. (2017a). Competent cells were transformed by electroporation (Eggeling and Bott, 2005) at 25 μF, 200 Ω, and 2.5 kV using plasmid DNA extracted from *E. coli* DH5α cells. Plasmid DNA extraction was performed with a GeneJET^TM^ Plasmid Miniprep Kit (Thermo Fisher Scientific, MA, United States) following the manufacturer’s information.

### Molecular Design and Genetic Engineering of *C. glutamicum* PUT Xyl and AVA Xyl

**FIGURE 1:**
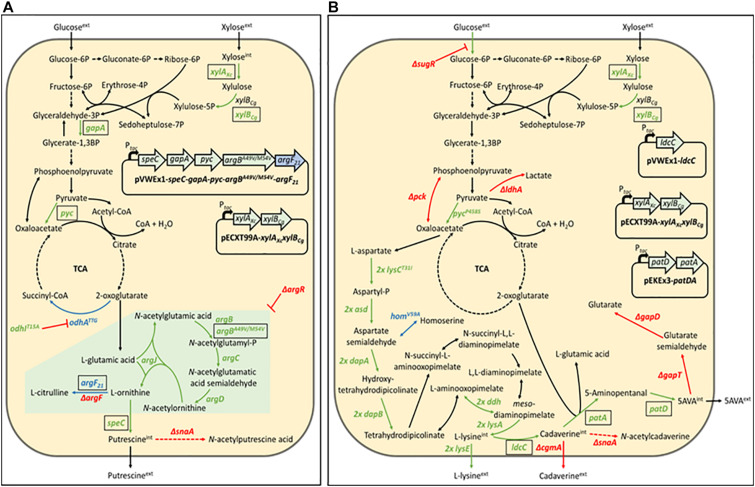
Schematic representation of the putrescine production route in *Corynebacterium glutamicum* PUT Xyl **(A)** and the 5-aminovalerate production route in *C. glutamicum* AVA Xyl **(B)**. Gene names of key enzymes are given next to the corresponding reaction (pointed arrows). Black-boxed gene names indicate plasmid-borne expression, unboxed gene names indicate genomic expression. Reaction routes involving more than one gene are depicted by dashed arrows. Repression is represented by blunt arrows. Upregulated reactions are shown in green, downregulated reactions are shown in blue, and deleted reactions are shown in red. The green-boxed arginine pathway in **(A)** indicate upregulation by deletion of the repressor of the arginine biosynthesis ***ArgR***. ***argB^*A4*9V/M54V^***, feedback-resistant N-acetyl glutamate kinase; ***argF***, ornithine transcarbamoylase; ***argF*_2_***_1_*, ornithine transcarbamoylase with reduced activity; ***argR***, repressor of the arginine biosynthesis; ***asd*** (2 copies), aspartate-semialdehyde dehydrogenase; ***cgmA***, cyclic beta-1,2-glucan modification protein; ***dapA*** (2 copies), 4-hydroxy-tetrahydrodipicolinate synthase; ***dapB*** (2 copies), 4-hydroxy-tetrahydrodipicolinate reductase; ***ddh*** (2 copies), meso-diaminopimelate D-dehydrogenase; ***gabD***, succinate-semialdehyde dehydrogenase; ***gabT***, 4-aminobutyrate aminotransferase; ***gapA***, Glyceraldehyde-3-phosphate dehydrogenase A; ***hom^*V5*9A^***, reduced activity homoserine dehydrogenase; ***ldcC***, constitutive lysine decarboxylase; ***ldhA***, D-lactate dehydrogenase; ***lysA*** (2 copies), diaminopimelate decarboxylase; ***lysC^*T31*1I^*** (2 copies), high activity aspartokinase; ***lysE*** (2 copies), lysine exporter; ***odhA*^*TTG*^**, reduced activity 2-oxoglutarate dehydrogenase; ***odhI^*T1*5A^***, high activity oxoglutarate dehydrogenase inhibitor; ***patA***, putrescine aminotransferase; ***patD***, gamma-aminobutyraldehyde dehydrogenase; ***pck***, phosphoenolpyruvate carboxykinase; ***P*_*tac*_**, IPTG-inducible tac promoter; ***pyc***, pyruvate carboxylase; ***pyc^*P45*8S^***, high activity pyruvate carboxylase; ***snaA***, N-acetyltransferase; ***speC***, ornithine decarboxylase; ***sugR***, central transcriptional regulator of the carbon metabolism; ***xylA*_*Xc*_**, xylose isomerase from *Xanthomonas campestris*; ***xylB*_*Cg*_**, xylulose kinase from *C. glutamicum.*

The *C. glutamicum* ATCC13032 derived strain NA6 (Nguyen et al., 2015) used for the construction of xylose-utilizing Put Xyl (see Figure 1A) carries several modifications for putrescine production, including modifications for the improvement of 2-oxoglutarate supply (overexpression of *gapA* and *pyc*, and reduced 2-oxoglutarate dehydrogenase activity due to *odhA*^*TTG*^ and *odhI^*T*15A^*), for the de-repression of the arginine biosynthesis by chromosomal deletion of *argR*, for the overexpression of a feedback-resistant N-acetyl glutamate kinase (*argB^*A*49V/M54V^*), as well as for the prevention of by-product formation by the chromosomal deletions of *argF* and *snaA*. To avoid costly arginine supplementation due to the resulting arginine auxotrophy, a plasmid addiction system was created by inserting the *argF*_21_ gene variant (Schneider et al., 2012), which encodes an ornithine transcarbamoylase with reduced activity, into the pVWEx1-derived (Peters-Wendisch et al., 2001) expression vector (Nguyen et al., 2015).

*C. glutamicum*, harboring xylulose kinase *xylB* but lacking xylose isomerase *xylA* for the first conversion step of the xylose isomerase pathway, is not capable of metabolizing xylose naturally. In this work, the NA6 strain (Nguyen et al., 2015) was transformed with the ITPG-inducible pECXT99A-*xylA_*Xc*_xylB_*Cg*_* vector (Jorge et al., 2017b) for the overexpression of the xylose isomerase pathway, containing *xylA* from *Xanthomonas campestris* SCC1758 and *xylB* from *C. glutamicum* ATCC13032.

The corresponding 5-aminovalerate producer strain 5-AVA3 (pECXT99A-*xylA_*Xc*_xylB_*Cg*_*) already constructed in the previous work of Jorge et al. (2017b) was renamed to AVA Xyl (see Figure 1B) in this study. 5-AVA3 is a derivative of the genome-reduced lysine producer strain GRLys1 (Unthan et al., 2015), which lacks three genomic prophage regions and the phosphoenolpyruvate carboxykinase gene *pck*. Introduction of point mutations into the genes *lysC*, *pyc*, and *hom*, and chromosomal duplication of the lysine pathway genes *lysC*, *asd*, *dapA*, *dapB*, *ddh*, *lysA*, and *lysE* resulted in an effective lysine producer strain. For the construction of the production strain 5-AVA3 (Jorge et al., 2017b) deleted the transcriptional regulator of the sugar metabolism *sugR* to improve the glucose uptake, *ldhA*, *snaA*, *cgmA*, and *gabTDP* to prevent the side-product formation of lactate, N-acetyl cadaverine, cadaverine, as well as glutarate, and introduced the expression vectors pVWEx1-*ldcC* and pEKEx3-*patDA* to establish 5-AVA formation from lysine.

Supplementary Figure 1 shows the agarose gel of PCR fragments to verify the transformation of *C. glutamicum* NA6 with pECXT99A*-xylA_*Xc*_B_*Cg*_* vector (expected fragment size: 3,312 bp) and Supplementary Table 1 shows the pECXT99A primers used.

### Quantification of Sugars and Amines in the Fermented Broth

The sugars and amines in the fermentation broth were analyzed by RP-HPLC. The quantification of sugars was performed on a Shimadzu UFLC system LC-20AT Prominance Liquid Chromatograph equipped with a Refractive Index Detector (Shimadzu RID-10A), an autosampler (SIL 20AC HT) and a Column oven (CTO-20ACV) operated at 80°C. Shimadzu Lab Solutions data management software was used. The monomeric sugars (xylose and glucose) were resolved using Rezex RPM Pb^+^ cation exchange monosaccharide column (300 × 7.5 mm, Phenomenex) with Milli Q water (Millipore) at a 0.60 mL/min isocratic flow rate and a 10 μL sample injection volume. Putrescine and 5-AVA were quantified using a Shimadzu UFLC system equipped with a fluorescence detector (RF 20A) at excitation and emission wavelengths of 348 and 450 nm, respectively. The samples were filtered through 0.22 μm filter membranes (PALL Lifesciences), buffered at a pH 10 using borate buffer (Agilent) and pre-derivatized with o-phthaldialdehyde [OPA] (Agilent). Putrescine and 5-AVA were resolved with Zorbax Eclipse AAA column (Agilent) using 40 mM sodium phosphate buffer (Agilent) and acetonitrile methanol-water in the ratio (45:45:10) in a gradient elution program.

### Authentication of Putrescine Production From Xylose by Engineered *C. glutamicum* PUT Xyl

Even though the 5-aminovalerate production from xylose by *C. glutamicum* AVA Xyl was already shown by Jorge et al. (2017b), the putrescine production from xylose by *C. glutamicum* PUT Xyl has to be confirmed. Therefore, a growth and production study using CGXII minimal medium was conducted and subsequent putrescine measurement via HPLC analysis was performed. Since a previous growth experiment showed a slow growth of PUT Xyl (μ = 0.05 ± 0.003) on xylose (data not shown), a control supplemented with minimum glucose of 0.5 g L^–1^ for supporting the initial growth rate was chosen. Growth was observed by measuring OD_600_, and samples for HPLC analysis were taken after 6, 24, and 72 h of cultivation.

### Simultaneous Utilization of Glucose and Xylose by the Engineered *C. glutamicum* Strains in CGXII Minimal Medium by Batch Fermentation

For the production experiments, CGXII medium was formulated with different combinations of glucose and xylose making the total sugar concentration 40 g L^–1^ and a pH of 7. Overnight cultures of the *C. glutamicum* strains in BHI medium were harvested by centrifugation at 5,000 rpm for 10 min, washed in minimal medium without sugar and inoculated to the CGXII minimal medium with an initial optical density at 600 nm (OD_600_) of 1.0. The growth of the cells was monitored by measuring the absorbance at 600 nm in a spectrophotometer (Tecan plate reader infinite 200 Pro, Switzerland). Samples were withdrawn at the desired interval of time and checked for sugar consumption and product formation as per standard protocols.

### Preparation of Rice Straw Hydrolysate (RSH)

Rice straw procured from the local markets was used for the study. Dried rice straw milled into a particle size of ≤ 1 mm was subjected to alkali pretreatment with a solid loading of 20 % (w/w) and NaOH loading of 2 % (w/v), at 121 °C for 1 h. The pretreated biomass slurry was allowed to cool at room temperature, neutralized with 10 N H_2_SO_4_, wet sieved and air-dried before enzymatic hydrolysis. Later, the wet sieved neutralized biomass was saccharified with commercial cellulase (Zytex India Limited, Mumbai) with an enzyme load of 20 FPUs/g of the substrate and a biomass loading of 10 % (w/w), at 50 °C, 180 rpm for 48 h. A pH of 4.8 was maintained using citrate buffer (0.5 M). The liquid fraction called the RSH was separated from the whole slurries by centrifugation and filter-sterilized using 0.22 (47 mm) μm filter membranes (Millipore, Massachusetts, United States) prior to fermentation.

### Utilization of RSH as a Sole Carbon Source by AVA Xyl and PUT Xyl

To study the potential of utilizing low-value sugars in the agro-residual wastes by the *Corynebacterium* biocatalysts, shake flask batch fermentations were carried out at 30 °C and 200 rpm. The production medium with RSH was formulated with 20 g L^–1^ (NH_4_)_2_SO_4_, 5 g L^–1^ Urea, 1 g L^–1^ K_2_HPO_4_, 1 g L^–1^ KH_2_PO_4_, 0.25 g L^–1^ MgSO_4_. 7H_2_O, 42 g L^–1^ MOPS, 0.02 mg L^–1^ biotin, 10 mg L^–1^ CaCl_2_ and necessary trace elements in RSH, adjusted to pH 7 using 10 N NaOH. Overnight grown seed cultures in the BHI medium were inoculated to RSH based production medium (100 mL) to attain an initial OD of 1.0 and incubated at 30 °C, in a rotary shaker (200 rpm). The growth pattern of both strains was determined by a spectrophotometer. The sugar utilization and the production of the two recombinant strains were estimated by HPLC.

All the experiments were performed in triplicates and all the data were expressed as the mean ± standard deviation of three independent replicates.

## Results and Discussion

### Validation of Biosynthesis of Putrescine by the Recombinant Strain PUT Xyl in CGXII Medium

**FIGURE 2:**
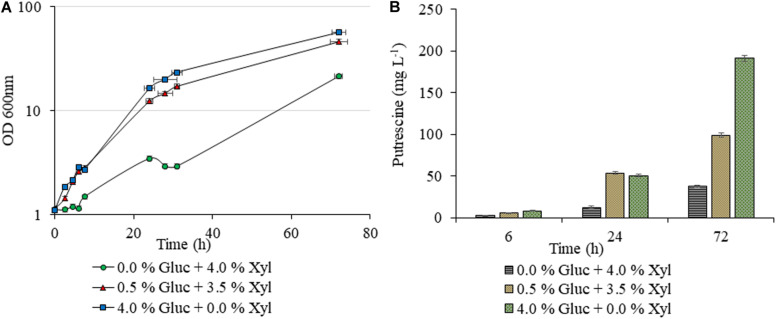
Putrescine production of *C. glutamicum* PUT Xyl. Growth curves of *C. glutamicum* PUT Xyl cultivated in shake flasks of 20 mL CGXII minimal medium supplemented with different compositions of glucose (Gluc) and xylose (Xyl) **(A)**, and putrescine concentrations measured after 0, 6, 24, and 72 h of cultivation **(B)**. Measurements are given as means from triplicates of independent cultivations with standard deviation.

Unlike AVA Xyl, which was studied earlier for 5-AVA biosynthesis, as indicated in the methodology, initially we checked the xylose utilization potentiality of the new construct PUT Xyl for proof of the concept. As shown in Figure 2B, a lower putrescine production of 26.66 ± 1.40 mg L^–1^ and a growth rate of 0.040 ± 0.002 h^–1^ was observed after 72 h, in the shake flask which initially contained 40 g L^–1^ of xylose alone as the carbon source. The growth was picked up to 0.137 ± 0.008 h^–1^ (Figure 2A) when a minimal amount of glucose (5 g L^–1^) was supplemented initially along with xylose (35 g L^–1^) and the putrescine production titer increased to 97.88 ± 2.07 mg L^–1^.

### Production of Putrescine and 5-AVA by PUT Xyl and AVA Xyl in CGXII Minimal Medium With the Simultaneous Utilization of Glucose and Xylose

**FIGURE 3:**
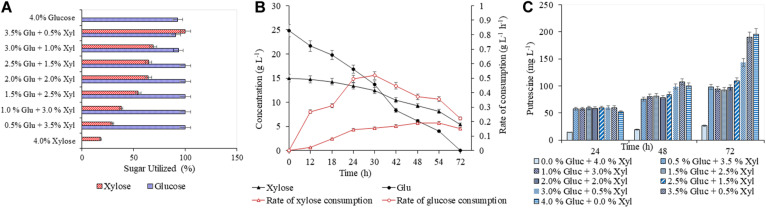
Production of putrescine in CGXII synthetic medium with different blends of glucose and xylose. The percentage utilization of glucose and xylose by PUT Xyl is shown in **(A)**. The glucose and xylose consumption rate of PUT Xyl is shown in **(B)**. The time profile of the putrescine production is shown in **(C)**. Measurements are given as means from triplicates of independent cultivations with standard deviation.

**FIGURE 4:**
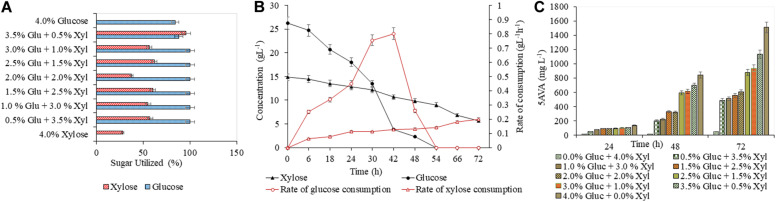
Production of 5-AVA in CGXII medium with different blends of glucose and xylose. The utilization of glucose and xylose by PUT Xyl is shown in **(A)**. The glucose and xylose consumption rate of AVA Xyl is shown in **(B)**. The time profile of the 5-AVA production is shown in **(C)**. Measurements are given as means from triplicates of independent cultivations with standard deviation.

A detailed study was conducted with varying concentrations of glucose (5–40 g L^–1^) and xylose (5–40 g L^–1^), to analyze the efficacy of both AVA Xyl and PUT Xyl strains in co-utilizing pure glucose and xylose initially making a total sugar of 40 g L^–1^ in CGXII minimal medium. After 72 h of fermentation, 96.74 ± 5.66 mg L^–1^ putrescine was obtained with a growth rate of 0.145 ± 0.100 h^–1^, when the PUT Xyl strain fermented an equal amount of glucose (20 g L^–1^) and xylose (20 g L^–1^). The same 1:1 combination of sugars yielded 602.74 ± 2.15 mg L^–1^ 5-AVA after 72 h and a growth rate of 0.187 ± 0.002 h^–1^ when AVA Xyl strain was used. The utilization of xylose by the two recombinant strains, even in the presence of glucose shows their efficacy to co-metabolize the xylose and glucose present in the fermentation medium. The xylose consumption was evidently facilitated once the glucose pool reached a depletion. This metabolic shift from glucose to xylose suggests that the xylose machinery including the overexpressed *xylA xylB* genes is also active even in the presence of glucose. When the medium contained 25 g L^–1^ glucose and 15 g L^–1^ xylose, the entire glucose and 64.28 ± 0.13% (Figure 3A) of xylose was consumed by PUT Xyl, which is about 9.29 ± 0.04 g L^–1^, at a rate of 0.152 ± 0.004 g L^–1^ h^–1^ (Figure 3B) to produce 109.43 ± 2.11 mg L^–1^ of putrescine (Figure 3C) after 72 h and grew faster (μ = 0.148 ± 0.060 h^–1^). The percentage of xylose utilized by the AVA Xyl strain is shown in Figure 4A. Almost 9.10 ± 0.05 g L^–1^ of xylose was uptaken by AVA Xyl at a rate of 0.198 ± 0.012 g L^–1^ h^–1^ (Figure 4B), along with the whole glucose, grew at a rate of 0.188 ± 0.005 h^–1^ and gave an AVA titer of 874.43 ± 0.98 mg L^–1^ (Figure 4C) after 72 h. The increase in the initial glucose concentration in the medium resulted in a slight decline of the xylose uptake which may be due to the repression offered by glucose.

A slight decrease in the production, of putrescine (92.93 ± 2.83 mg L^–1^) and 5-AVA (553.93 ± 4.67 mg L^–1^) as well as in the growth of PUT Xyl and AVA Xyl was observed with a combination of 15 g L^–1^ glucose and 25 g L^–1^ xylose. As mentioned in section “Validation of Biosynthesis of Putrescine by the Recombinant Strain PUT Xyl in CGXII Medium,” the least putrescine (26.66 ± 1.40 mg L^–^1) and also 5-AVA production (52.66 ± 0.09 mg L^–1^) was observed when xylose (40 g L^–1^) alone was the only sugar source. Though the highest amounts of putrescine of about 195.71 ± 0.69 mg L^–1^ and 5-AVA of 1508.71 ± 3.21 mg L^–1^ were obtained in the shake flask with 40 g L^–1^ glucose, significant productions were observed when glucose is partially replaced with different ratios of xylose. The comparable production titer of the respective compounds when a mixture of glucose and xylose was used, revealed that both the recombinant strains were capable of co utilizing them to produce respective products, the putrescine and 5-AVA.

### Exploitation of the Fermentable Sugars in the RSH for the Production of Putrescine and 5-AVA

**FIGURE 5:**
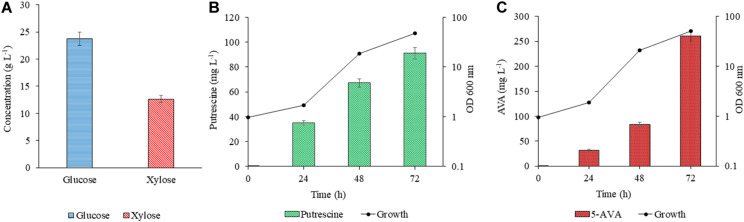
Concentration of glucose and xylose in RSH **(A)**. Production of putrescine **(B)** and 5 AVA **(C)** from RSH. Hydrolysate contained 2.37 g L^–1^ glucose + 12.6 g L^–1^ xylose. The downward diagonal column represents the production and the line graph with marker shows the absorbance at OD 600 nm. Measurements are given as means from triplicates of independent cultivations with standard deviation.

The efficacy of PUT Xyl and AVA Xyl strains to ferment the saccharified sugars in the lignocellulosic hydrolysate was evaluated after validating the proof of concept in the CGXII synthetic medium. The RSH after 48 h of hydrolysis contained glucose (23.76 g L^–1^) and xylose (12.65 g L^–1^) (Figure 5A) and the formulated RSH based production medium had a total sugar (mainly glucose and xylose) of 3.64 %. The sugar utilization, growth and production by PUT Xyl and AVA Xyl in the RSH based medium containing (23.7 g L^–1^ glucose + 12.6 g L^–1^ xylose) was comparable to that in the CGXII medium, with the most proximate sugar concentration (25 g L^–1^ glucose and 15 g L^–1^ xylose). However, engineered *C. glutamicum* strains when cultivated in the RSH based medium had a longer lag phase. After 24 h, the PUT Xyl strain attained a growth rate of 0.013 ± 0.004 h^–1^, whereas the AVA Xyl strain had a growth rate of 0.003 ± 0.001 h^–1^. Thereafter, the growth was picked up to 0.130 ± 0.028 h^–1^ and 0.140 ± 0.031 h^–1^ by PUT Xyl and AVA Xyl, respectively, and the xylose and glucose in the RSH were fermented to yield 91.00 ± 2.58 mg L^–1^ putrescine (Figure 5B) and 260.33 ± 2.47 mg L^–1^ 5-AVA (Figure 5C) after 72 h.

Polyamide (PA), commonly known as nylon, is a polymer with a myriad of pharmaceutical and industrial applications and is currently synthesized by petrochemical routes. Chemically, the polymer backbone is composed of repetitive units of diamines and dicarboxylic acids that contain different numbers of carbon atoms, imparting a variety of material properties. Globally, the demand for polyamides has fairly increased and is expected to grow at a CAGR of 7.1 % in a forecast period of 2020–2027. The petroleum-based fossil fuels remarkably contribute to 60 % of one-fifth of the CO_2_ emission because nearly 82 % of the global energy needs are met by non-renewable energy sources such as petroleum, coal and natural gas (Gupta and Verma, 2015).

Biomass has been considered to be the only sustainable source of organic carbon on earth and the net-zero carbon emission makes it the best analogy to petroleum for the production of value-added chemicals (Sharma et al., 2019). Howbeit we have employed the plasmid-based expression, a genome-based expression for the amplification of the *xylA* and *xylB*, genes are preferred over the plasmid-based expression system to avert its instability problems for industrial-scale applications. In the current study, we have focused on the bacterial synthesis of a diamine and a dicarboxylic acid by exploiting the low-value sugars in the lignocellulosic biomass—rice straw, using the engineered *C. glutamicum* strains capable of consuming xylose and glucose. This was established by genetically modifying the metabolic pathways in *C. glutamicum* and make it a self-sufficient host for the utilization of the C5 sugar xylose, a fair amount of which is gone underutilized in the form of agro-residues. Since the wild type *C. glutamicum* deficient of the xylose isomerase activity is unable to utilize xylose neither in aerobic nor in oxygen-deprived conditions (Kawaguchi et al., 2006) here in this study, we have further modified the NA6 strain, previously reported by Nguyen et al. (2015), for xylose utilization and its biotransformation to putrescine. Meiswinkel et al. (2013) studied the growth of *C. glutamicum* strains harboring different *xylA* and *xylB* genes from *B. subtilis, E. coli, M. smegmatis*, and *X. campestris* and reported that the recombinant strains with heterologous expression of the endogenous *xylB* gene from *C. glutamicum* in addition to the *xylA* gene from *X. campestris* improved xylose utilization and grew well (μ = 0.199 ± 0.009 h^–1^) in CGXII medium with xylose as the sole carbon source. The toxicity of putrescine to any microorganisms (Incorvia, 2015) was believed to be a hindrance for the active exploration of bacterial putrescine production (Qian et al., 2009) but *C. glutamicum* can tolerate higher concentrations (44 g L^–1^) of putrescine (Schneider and Wendisch, 2010; Jorge et al., 2017a). An *E. coli* strain XQ52 harboring p15SpeC was reported to produce 21.7 g L^–1^ in 32 h, by the consumption of glucose via fed-batch fermentation (Noh et al., 2017). In our study, the putrescine titer obtained using the engineered *C. glutamicum* strain PUT Xyl was 109.43 ± 2.11 mg L^–1^ in the shake flasks with CGXII containing 25 g L^–1^ glucose and 15 g L^–1^ xylose.

The collection of raw material, its availability throughout the seasons, transportation and storage are the major difficulties in any study related to the agro residual biomass. The digestibility of cellulose present in lignocellulosic biomass is hindered by many physicochemical, structural, and compositional factors are one of the major concerns. The biomass needs to be treated so that the cellulose in the plant fibers is exposed. The goal of the pretreatment process is to break down the lignin structure and disrupt the crystalline structure of cellulose so that the enzymes can easily access and hydrolyze the cellulose. The alkali pretreatment decreases the lignin by about 47 % and increases the glucan by about 50 % (Oberoi et al., 2012). The enzymatic hydrolysis of the alkali pretreated rice straw had a fair glucose (23.7 g L^–1^) and xylose (12.6 g L^–1^) composition, which were fermented to value-added chemicals such as 5-AVA (260.33 ± 2.47 mg L^–1^) and putrescine (91.00 ± 2.58 mg L^–1^) by AVA Xyl and PUT Xyl. The recombinant *C. glutamicum* strains overexpressing the transformed *xylA*, *xylB* genes were proved efficient to uptake the fermentable sugars from RSH based medium and biosynthesize the compounds of interest. The recombinant *C. glutamicum* strains co-utilize the xylose along with glucose present in the lignocellulosic hydrolysates but the presence of pre-treatment derived inhibitors like furfural and/or hydroxyl methyl furfural attributes to the slower growth and production when hydrolysate based media is used (Gopinath et al., 2011). In this study, we have observed the utilization of both glucose and xylose by the modified *C. glutamicum* strains in the CGXII medium and as well as in the RSH based medium. A higher titer of 5-AVA (39.93 g L^–1^) than from the glucose fermentation was obtained in Miscanthus hydrolysate solution by fed-batch fermentation employing the recombinant *C. glutamicum* KCTC 1857 expressing the *davBA* genes (Joo et al., 2017). Industrial sugar beet thick juice was reported as a suitable carbon source for *Corynebacterium* and produced putrescine with a volumetric productivity of 0.28 ± 0.01 g L^–1^ h^–1^ (Meiswinkel et al., 2014). In our study, *C. glutamicum* PUT Xyl produced putrescine from the rice straw hydrolysate-based medium with a volumetric productivity of 1.26 ± 0.31 mg L^–1^ h^–1^. A study by Nguyen and Lee (2019), represented the bioconversion of methane to putrescine using an engineered *M*e*thylomicrobium alcaliphilum* 20ZE4-pACO strain with a putrescine titer of 98.08 mg L^–1^. A lower growth rate was observed in RSH based medium when compared to the most proximate sugar concentration of that in the CGXII minimal medium for both the strains, which may be due to the hindrances offered by the pre-treatment derived inhibitors (Gopinath et al., 2011).

## Conclusion

The ability for the simultaneous utilization of glucose and the pentose sugar xylose by the two engineered *C. glutamicum* strains AVA Xyl and PUT Xyl to produce 5-AVA and putrescine opened up a new avenue to utilize them for the production of such value-added products from renewable sources such as lignocellulosic biomass which generally goes as a surplus waste product in the agricultural sector. The tolerance of *C. glutamicum* to grow in pretreated biomass hydrolysate is yet another added advantage of this wonderful industrial strain making them an excellent biocatalyst in biorefineries for improving the techno-economic feasibility of the entire processes. As of now, we could demonstrate the production of value-added wide spectrum products such as lysine, xylitol, xylonic acid, etc., from biomass using carefully drafted and engineered *C. glutamicum* strains. Here we have demonstrated a green bioprocess for the production of two highly demanded building blocks of the polyamide industry such as 5AVA and putrescine from a renewable raw material like rice straw. Process strategies employing synthetic, mutually dependent consortia (Sgobba et al., 2018) or dynamically controlled co-cultivation of two recombinant *C. glutamicum* strains (Pérez-Garcia et al., 2021) may be used for further optimization. Successful upscaling of the process can be a breakthrough in white biotechnology for the production of bioplastics.

## Data Availability Statement

The raw data supporting the conclusions of this article will be made available by the authors, without undue reservation.

## Author Contributions

KN and VW acquired funding and designed the study. KS carried out the biomass pretreatment, shake flask fermentation, and production data analysis. SH and VW constructed the production strains as part of the Indo German collaboration work. SH wrote the manuscript portions pertaining to the strain construction details. KS and KN designed and drafted the final manuscript. VW did the critical reading. All authors read and approved the final manuscript.

## Conflict of Interest

The authors declare that the research was conducted in the absence of any commercial or financial relationships that could be construed as a potential conflict of interest.
