# Corrigendum: Production of biopolyamide precursors 5-amino valeric acid and putrescine from rice straw hydrolysate by engineered *Corynebacterium glutamicum*

**DOI:** 10.3389/fbioe.2025.1588115

**Published:** 2025-03-24

**Authors:** Keerthi Sasikumar, Silvin Hannibal, Volker F. Wendisch, K. Madhavan Nampoothiri

**Affiliations:** ^1^ Microbial Processes and Technology Division (MPTD), CSIR-National Institute for Interdisciplinary Science and Technology, Thiruvananthapuram, India; ^2^ Academy of Scientific and Innovative Research (AcSIR), Ghaziabad- 201002, India; ^3^ Genetics of Prokaryotes, Faculty of Biology & CeBiTec, Bielefeld University, Bielefeld, Germany

**Keywords:** 5-amino valeric acid, *Corynebacterium glutamicum*, polyamides, putrescine, rice straw hydrolysate

In the published article, there was an error in **Affiliation 2**. Instead of “^2^ Academy of Scientific and Innovative Research (AcSIR), Ghaziabad, India,” it should be “^2^ Academy of Scientific and Innovative Research (AcSIR), Ghaziabad- 201002, India.”

The authors apologize for this error and state that this does not change the scientific conclusions of the article in any way. The original article has been updated.

