# Supplementary material for: Production of Biopolyamide Precursors 5-Amino Valeric Acid and Putrescine From Rice Straw Hydrolysate by Engineered Corynebacterium glutamicum
Source: Front Bioeng Biotechnol. 2021 Mar 29;9:635509. doi: 10.3389/fbioe.2021.635509 (PMC8044859; doi:10.3389/fbioe.2021.635509)
Supplement: Supplementary file 1 [file Data_Sheet_1.docx]

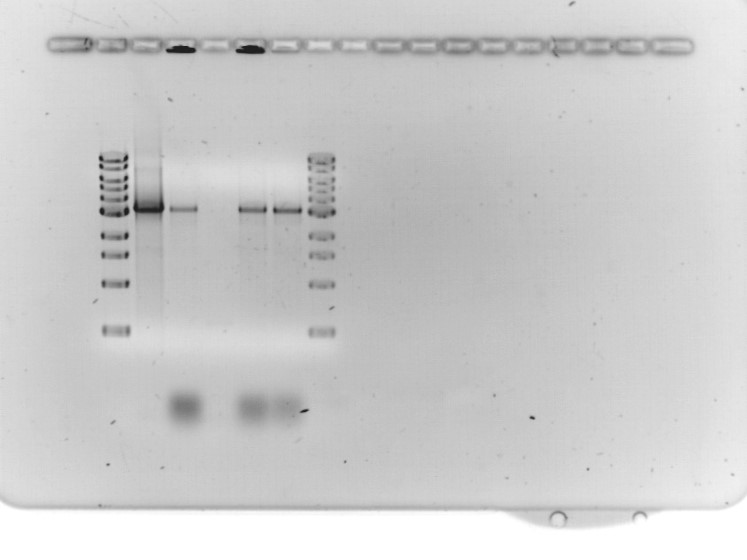


**1 2 3 4 5 6 7**

**10**

**6**

**4**

**3**

**2**

**1.5**

**1**

**0.5**

**10**

**6**

**4**

**3**

**2**

**1.5**

**1**

**0.5**

**8**

**5**

**8**

**5**

**kbp kbp**

**Supplementary figure 1**

**1 % agarose gel of PCR fragments to verify transformation of *C. glutamicum* NA6 with pECXT99A*-xylA_Xc_B_Cg_* vector (expected fragment size: 3312 bp).**

The PCR was performed with ALLin™ HiFi polymerase (HighQu) using the manufacturer's protocol, primers pECXT-fw and pECXT-rv and different templates (lanes 2 – 6). 5 µL PCR samples were mixed with 1 µL loading dye and transferred to an 1 % agarose gel. Agarose gel electrophoresis was performed using TAE running buffer, and a tension of 100 V. The gel was incubated for 3 min in ethidiumbromide, and a picture was taken at an UV transilluminator. **Lane 1 and 7:** size standard (NEB 1 kb DNA Ladder); **Lane 2:** 20 ng pECXT99A*-xylA_Xc_B_Cg_* vector DNA; **Lane 3:** E. coli DH5α (pECXT99A*-xylA_Xc_B_Cg_*) colony; **Lane 4:** no template (negative control); **Lane 5:** AVA Xyl colony; **Lane 6:** PUT Xyl colony.

**Supplementary table 1: Primer list**

| **Primer name** | **5’ 🡪 3’ DNA sequence** | **Description** |
| --- | --- | --- |
| pECXT-fw | AATACGCAAACCGCCTCTCC | pECXT99A vector forward primer |
| pECXT-rv | TACTGCCGCCAGGCAAATTC | pECXT99A vector reverse primer |
